# Supplementary material for: Metabolic Fingerprinting of Muscat of Alexandria Grape Musts during Industrial Alcoholic Fermentation Using HS-SPME and Liquid Injection with TMS Derivatization GC-MS Methods
Source: Molecules. 2023 Jun 8;28(12):4653. doi: 10.3390/molecules28124653 (PMC10305144; doi:10.3390/molecules28124653)
Supplement: Supplementary file 1 [file molecules-28-04653-s001.zip › molecules-2419277-supplementary.pdf]

Metabolic Fingerprinting of Muscat of Alexandria Grape Musts during Industrial Alcoholic Fermentation Using HS-SPME and Liquid Injection with TMS Derivatization GC-MS Methods

Maria Marinaki[1,2,3], Thomai Mouskeftara[2,3,4], Panagiotis Arapitsas[5,6], Kyriaki Zinoviadou[7], and Georgios Theodoridis[1,2,3]

[1] Laboratory of Analytical Chemistry, School of Chemistry, Aristotle University of Thessaloniki, 54124 Thessaloniki, Greece
[2] BiomicAUTh, Center for Interdisciplinary Research and Innovation (CIRI-AUTH), 57001 Thessaloniki, Greece
[3] FoodOmicsGR Research Infrastructure, AUTh Node, Center for Interdisciplinary Research and Innovation (CIRI-AUTH), 57001 Thessaloniki, Greece
[4] Department of Medicine, Aristotle University, 54124 Thessaloniki, Greece
[5] Department of Wine, Vine and Beverage Sciences, School of Food Science, University of West Attica, 12243 Athens, Greece
[6] Research and Innovation Centre, Fondazione Edmund Mach, 38010 Trento, Italy
[7] American Farm School, Perrotis College, 57001 Thessaloniki, Greece; kzinov@afs.edu.gr

Table S1. Tank number and volume, winery, starting and ending date of fermentation, temperature of fermentation, Baume and acidity the first day of fermentation, concentration of sugars the last day of fermentation, alcohol content, days of oxygenation and code numbers of the analyzed samples with the proposed methods

Tank	Winery	Year	Tank Volume (L)	Starting date	Ending date	Fermentation Temperature (°C)	Baume	Acidity (gL^{-1})	Sugars at the end of fermentation (gL^{-1})	Alcohol content (%vol)	Oxygenation days
**1**	1	2019	20000	05/09[a]	21/09	16.0	12.5	5.5	<2	13	3rd
**2**	1	2019	20000	05/09	18/09	16.0	12.6	5.5	<2	13.1	3rd
**3**	1	2019	20000	06/09	19/09	16.0	12.4	5.4	<2	13	3rd
**8**	2	2019	15000	06/09	20/09	17.8	12.1	5.8	0.9	12.4	2nd and 3rd
**10**	2	2019	15000	06/09	20/09	17.8	12.5	5.7	<1	12.6	2nd and 3rd
**11**	2	2019	15000	06/09	20/09	17.8	11.9	6.0	1	12.3	2nd and 3rd
**73**	3	2019	45400	04/09	17/09	17.0	12.1	6.1	<2	12.6	2nd
**75**	3	2019	43800	04/09	17/09	17.0	12.3	5.9	<2	12.8	2nd
**96**	3	2019	27150	12/09	26/09	17.0	12.8	5.6	<2	13.3	2nd
**75_1**	3	2020	44600	10/09	23/09	17.5	12.5	5.7	<2	13.1	3rd and 8th
**95_1**	3	2020	28400	10/09	23/09	17.0	12.4	5.5	<2	12.9	3rd and 8th

[a] The codes of samples express the tank number and the date of sampling (e.g., 7304 = tank 74, sampling 04/09 or 95231= tank 95_1, sampling 23/09, *the numbers 09 of the date are not shown because all sampling dates are in September)

Table S2. Identified metabolites with HS-SPME-GC-MS method, their chemical formula, retention time (RT), experimental and bibliographic retention index (RI_{exp} and RI_{lit}) and their relative concentration (mg L^{-1} ± std) the 1st, 5th, 8th and 13th day of fermentation

Compound name	Chemical Formula	RT[a]	RI_{exp}[b]	RI_{lit}[c]	Concentration (mg L^{-1} ± std)			
					1st day	5th day	8th day	13th day
				**ACETATES**				
**Ethyl acetate**	C4H8O2	1.73	679	621[d]	0.41±0.60	2.73±0.89	4.18±1.01	4.29±0.77
**Propyl acetate**	C5H10O2	2.77	725	705	0.00±0.00[e]	0.01±0.00	0.02±0.00	0.02±0.00
**Isobutyl acetate**	C6H12O2	3.83	769	771	0.03±0.05	0.19±0.06	0.25±0.05	0.24±0.04
**Isoamyl acetate**	C7H14O2	6.14	868	862	1.25±2.73	7.37±2.93	10.06±3.84	8.47±4.35
**2-Methylbutyl acetate**	C7H14O2	6.18	872	866	0.10±0.19	0.70±0.35	1.03±1.09	0.66±0.37
**Amyl acetate**	C7H14O2	7.01	903	908	0.00±0.01	0.03±0.02	0.07±0.02	0.09±0.02
**3-Methylpentyl acetate**	C8H16O2	8.58	972	988	0.01±0.01	0.03±0.01	0.05±0.01	0.05±0.01
**Hexyl acetate**	C8H16O2	9.27	1001	1011	0.44±0.80	1.14±0.33	0.94±0.31	0.73±0.25
**Octyl acetate**	C10H20O2	13.40	1208	1211	0.01±0.01	0.02±0.00	0.02±0.00	0.02±0.00
**Phenethyl acetate**	C10H12O2	14.19	1247	1246	1.21±2.46	7.76±3.20	7.91±3.05	6.12±2.72
***alpha*-Terpinyl acetate**	C12H20O2	14.26	1250	1214	0.01±0.01	0.06±0.04	0.06±0.03	0.04±0.02
**Farnesyl acetate**	C17H28O2	21.96	1813	1818	0.00±0.00	0.00±0.00	0.01±0.00	0.01±0.01
				**FATTY ACIDS**				
**Butanoic acid**	C4H8O2	8.63	977	844	0.35±0.52	1.61±0.35	1.34±0.31	1.09±0.27
**Hexanoic acid**	C6H12O2	8.70	986	989	0.34±0.51	1.59±0.35	1.32±0.31	1.07±0.27
**Octanoic acid**	C8H16O2	12.80	1190	1174	3.30±5.74	13.37±2.69	13.02±2.74	10.8±1.91
**Nonanoic acid**	C9H18O2	14.33	1254	1278	0.08±0.05	0.14±0.05	0.08±0.02	0.07±0.02
***n*-Decanoic acid**	C10H20O2	16.03	1362	1363	2.20±3.76	6.86±2.54	4.46±1.44	3.37±0.76
**Dodecanoic acid**	C12H24O2	18.64	1540	1567	0.03±0.06	0.13±0.05	0.09±0.04	0.08±0.05
				**ALCOHOLS**				
**2-Methylpropan-1-ol**	C4H10O	1.83	684	697	0.10±0.14	0.61±0.23	0.87±0.12	1.02±0.22
**Isoamyl alcohol**	C5H12O	3.14	741	741	1.67±2.97	8.54±2.84	10.35±1.95	10.41±2.26
**2-Methylbutan-1-ol**	C5H12O	3.24	743	745	0.43±0.71	2.72±1.24	3.03±0.64	3.29±0.97
**2,3-Butanediol**	C4H10O2	4.09	780	789	0.03±0.06	0.42±0.19	0.96±0.19	1.04±0.16
**3-Methylpentan-1-ol**	C6H14O	5.44	838	829	0.00±0.01	0.02±0.01	0.03±0.01	0.03±0.01
**3-Hexen-1-ol**	C6H12O	5.65	847	853	0.05±0.01	0.03±0.01	0.03±0.01	0.02±0.00
**1-Hexanol**	C6H14O	5.97	860	867	0.65±0.15	0.35±0.12	0.26±0.10	0.26±0.11
**1-Octen-3-ol**	C8H16O	8.55	968	982	0.04±0.01	0.04±0.01	0.04±0.01	0.04±0.01
**6-Methylhept-5-en-2-ol**	C8H16O	8.86	983	979	0.01±0.00	0.01±0.00	0.00±0.00	0.00±0.00
**2,6-Dimethyl-3,7-octadiene-2,6-diol**	C10H18O2	9.12	995	1176	0.03±0.04	0.09±0.03	0.07±0.02	0.06±0.02
**Benzyl alcohol**	C7H8O	9.89	1031	1032	0.00±0.00	0.00±0.00	0.00±0.00	0.00±0.00
**2-Phenylethanol**	C8H10O	11.59	1108	1110	2.72±4.71	12.35±3.61	13.24±3.37	12.42±3.26

**2,6-dimethylocta-1,7-diene-3,6-diol**	C10H18O2	13.97	1233	1265	0.02±0.02	0.11±0.03	0.13±0.03	0.11±0.02
**1-Decanol**	C10H22O	14.42	1259	1269	0.01±0.00	0.02±0.00	0.03±0.01	0.03±0.00
**1-Dodecanol**	C12H26O	17.49	1457	1419	0.04±0.03	0.15±0.05	0.14±0.02	0.12±0.03
**2,2-Dimethyloctan-1-ol**	C10H22O	17.79	1474	-	0.00±0.00	0.01±0.00	0.01±0.00	0.01±0.00
**2,4-Ditert-butylphe-nol**	C14H22O	17.95	1488	1512	0.24±0.16	0.84±0.61	0.83±0.49	0.61±0.56
				**ALDEHYDES**				
**3-Methylbutanal**	C5H10O	2.09	691	<800	0.02±0.02	0.05±0.01	0.06±0.02	0.08±0.02
**2-Methylbutyralde-hyde**	C5H10O	2.18	699	661	0.00±0.00	0.00±0.00	0.00±0.00	0.00±0.00
**Hexanal**	C6H12O	4.41	794	800	0.03±0.02	0.01±0.00	0.01±0.01	0.01±0.00
**Benzaldehyde**	C7H6O	8.23	959	959	0.04±0.04	0.03±0.02	0.01±0.00	0.01±0.00
**Benzeneacetalde-hyde**	C8H8O	10.09	1039	1042	0.00±0.00	0.00±0.00	0.00±0.00	0.00±0.00
**Octadecanal**	C18H36O	20.36	1670	2017	0.00±0.00	0.01±0.00	0.01±0.00	0.01±0.00
				**C-13 NORISOPRENOIDS**				
***alpha*-Ionone**	C13H20O	14.64	1275	-	0.01±0.01	0.02±0.01	0.01±0.00	0.01±0.00
***beta*-Ionone**	C13H20O	14.86	1288	1294	0.03±0.02	0.05±0.02	0.03±0.01	0.02±0.01
***beta*-Damascenone**	C13H18O	16.20	1369	1373	0.36±0.36	0.59±0.22	0.26±0.11	0.20±0.08
				**ESTERS**				
**Isobutyl lactate**	C7H14O3	2.76	710	718	0.07±0.13	0.71±0.27	1.51±0.26	1.66±0.28
**Methyl hexanoate**	C7H14O2	7.25	915	922	0.00±0.00	0.01±0.00	0.01±0.00	0.00±0.00
**Isobutyl hexanoate**	C10H20O2	12.19	1136	1136	0.00±0.00	0.00±0.00	0.00±0.00	0.00±0.00
**Propyl octanoate**	C11H22O2	14.71	1279	1247	0.00±0.00	0.01±0.00	0.01±0.00	0.01±0.00
**2-Methylpentyl 2-methylpentanoate**	C12H24O2	14.97	1291	-	0.00±0.00	0.02±0.01	0.03±0.01	0.03±0.01
**Propyl decanoate**	C13H26O2	17.68	1471	1473	0.00±0.00	0.01±0.00	0.00±0.00	0.00±0.00
**Methyl Dodecano-ate**	C13H26O2	18.14	1500	1518	0.01±0.02	0.02±0.02	0.00±0.00	0.00±0.00
**Isobutyl decanoate**	C14H28O2	18.42	1523	1549	0.00±0.00	0.01±0.00	0.01±0.00	0.01±0.00
**3-Methylbutyl Deca-noate**	C15H30O2	19.71	1623	1615	0.01±0.02	0.07±0.04	0.12±0.09	0.17±0.11
**2-Phenylethyl hexa-noate**	C14H20O2	19.79	1629	1645	0.02±0.03	0.08±0.03	0.07±0.05	0.04±0.03
**Diethyl nonanedi-oate**	C13H24O4	20.22	1651	1652	0.00±0.00	0.01±0.00	0.01±0.00	0.01±0.00
**3-Methylbutyl do-decanoate**	C17H34O2	22.06	1819	1844	0.00±0.00	0.01±0.01	0.02±0.02	0.03±0.02
				**ETHYL ESTERS**				
**Ethyl butyrate**	C6H12O2	4.39	793	801	0.02±0.03	0.12±0.04	0.20±0.06	0.19±0.07
**Ethyl hexanoate**	C8H16O2	8.97	988	984	0.42±0.78	3.44±1.31	3.87±0.96	3.27±1.06
**Ethyl heptanoate**	C9H18O2	11.12	1088	1095	0.00±0.00	0.01±0.00	0.01±0.00	0.01±0.00
**Ethyl octanoate**	C10H20O2	13.08	1194	1183	1.40±2.62	9.45±3.26	13.23±2.13	12.3±2.88
**Ethyl nonanoate**	C11H22O2	14.77	1285	1286	0.02±0.03	0.06±0.12	0.02±0.00	0.02±0.00

**Ethyl 3-hydroxyhex-anoate**	C8H16O3	15.38	1341	1126	0.00±0.00	0.01±0.00	0.00±0.00	0.00±0.00
**Ethyl decanoate**	C12H24O2	16.32	1377	1380	0.84±1.51	4.97±3.02	6.68±1.81	7.35±3.08
**Ethyl 3-hydroxy-tridecanoate**	C15H30O3	18.32	1517	1539	0.00±0.00	0.01±0.01	0.01±0.01	0.02±0.02
**Ethyl Dodecanoate**	C14H28O2	19.06	1572	1590	0.11±0.21	0.77±0.38	1.47±0.91	1.94±1.37
**Ethyl tetradecanoate**	C16H32O2	21.48	1768	1993	0.01±0.02	0.08±0.05	0.12±0.06	0.10±0.08
**Ethyl 9-hexadeceno-ate**	C18H34O2	23.46	1946	1977	0.00±0.00	0.02±0.01	0.03±0.02	0.04±0.03
**Ethyl hexadecanoate**	C18H36O2	23.66	1965	1978	0.01±0.01	0.14±0.05	0.20±0.07	0.26±0.11
**TERPENES**								
**Limonene oxide**	C10H16O	7.60	930	1109	0.01±0.00	0.02±0.01	0.04±0.01	0.05±0.01
***beta*-Myrcene**	C10H16	8.73	980	989	0.44±0.14	0.46±0.12	0.45±0.07	0.41±0.09
***o*-Cymene**	C10H14	9.57	1018	1022	0.07±0.02	0.09±0.03	0.09±0.01	0.09±0.02
**D-Limonene**	C10H16	9.65	1024	1028	0.31±0.12	0.50±0.16	0.48±0.12	0.44±0.15
***trans-beta*-Ocimene**	C10H16	9.77	1027	1036	0.12±0.04	0.16±0.04	0.15±0.02	0.13±0.03
***cis-beta*-Ocimene**	C10H16	10.00	1035	1034	0.20±0.07	0.28±0.07	0.25±0.04	0.23±0.06
***gamma*-Terpinene**	C10H16	10.29	1048	1063	0.02±0.01	0.04±0.01	0.03±0.01	0.03±0.01
***trans*-Linalool oxide**	C10H18O2	10.57	1054	1072	0.05±0.02	0.04±0.01	0.02±0.01	0.02±0.01
**4-Carene**	C10H16	10.76	1074	1014	0.00±0.00	0.00±0.00	0.00±0.00	0.00±0.00
**Linalool**	C10H18O	11.20	1090	1100	3.70±1.26	5.01±1.05	3.24±0.75	2.54±0.39
**Ho-trienol**	C10H16O	11.31	1095	1107	1.29±0.45	1.05±0.26	0.66±0.14	0.52±0.11
***trans*-Rose oxide**	C10H18O	11.41	1100	1117	0.01±0.00	0.02±0.01	0.03±0.02	0.02±0.01
**Cosmene**	C10H14	11.82	1122	1130	0.06±0.03	0.14±0.05	0.12±0.03	0.10±0.03
***trans*-Alloocimene**	C10H16	12.01	1127	1128	0.03±0.01	0.02±0.01	0.02±0.00	0.02±0.01
**Nerol oxide**	C10H16O	12.26	1142	1151	0.08±0.04	0.14±0.04	0.09±0.02	0.07±0.02
***trans*-2-Pinanol**	C10H18O	12.47	1153	1121	0.06±0.12	0.56±0.17	0.83±0.15	0.78±0.21
***trans*-Ocimenol**	C10H18O	12.53	1184	1132	0.01±0.01	0.03±0.01	0.01±0.00	0.01±0.01
***alpha*-Terpineol**	C10H18O	13.17	1197	1193	1.30±0.58	1.99±0.53	1.02±0.28	0.83±0.28
**Nerol methyl ether**	C11H20O	13.51	1211	1211	0.02±0.01	0.05±0.01	0.04±0.01	0.03±0.01
**Citronellol**	C10H20O	13.63	1218	1226	0.22±0.06	0.34±0.09	0.28±0.09	0.24±0.05
**2-Bornene**	C10H16	13.76	1224	1239	0.00±0.00	0.01±0.00	0.01±0.01	0.01±0.01
**Geraniol**	C10H18O	14.07	1239	1232	0.74±0.17	0.84±0.21	0.45±0.16	0.35±0.08
**Nerol**	C10H18O	14.54	1266	1253	0.06±0.12	0.62±0.23	0.84±0.17	0.78±0.24
**OTHERS**								
**2-Methyl-1,3-Penta-diene**	C6H10	1.92	690	635	0.01±0.00	0.02±0.00	0.02±0.00	0.02±0.00
**1-Ethoxy-1-methoxy-ethane**	C5H12O2	2.11	696	641	0.00±0.00	0.01±0.01	0.01±0.00	0.01±0.00
**Pentane-2,3-dione**	C5H18O2	2.55	715	675	0.01±0.02	0.10±0.04	0.19±0.03	0.22±0.04
**2,4,5-Trimethyl-1,3-dioxolane**	C6H12O2	2.97	732	745	0.02±0.03	0.22±0.12	0.30±0.06	0.29±0.06
**1,1-Diethoxy-3-methylbutane**	C9H20O2	7.82	939	948	0.00±0.00	0.00±0.00	0.01±0.00	0.00±0.00
**Tricyclo [4,2,2,0 (1,5)] dec-7-ene**	C10H14	7.93	942	-	0.01±0.00	0.01±0.00	0.01±0.00	0.01±0.00

**Mesitylene**	C_9H_{12}	8.29	961	970	0.18±0.05	0.25±0.02	0.27±0.02	0.28±0.01
**1-Methylidene-4-prop-1-en-2-ylcyclohexane**	$C_{11}H_{16}$	9.37	1006	1003	0.02±0.01	0.03±0.01	0.03±0.00	0.03±0.01
**2-Butan-2-ylcyclopentan-1-one**	$C_9H_{16}O$	9.47	1019	1128	0.00±0.00	0.00±0.00	0.00±0.00	0.00±0.00
**3-Ethenyl-1,2-dimethylcyclohexa-1,4-diene**	$C_{10}H_{16}$	10.75	1069	-	0.01±0.00	0.01±0.00	0.01±0.00	0.01±0.00
**2-Ethenyl-1,3-dimethylbenzene**	$C_{10}H_{14}$	11.03	1081	1070	0.07±0.02	0.12±0.03	0.13±0.02	0.12±0.03
**2,6-Dimethylocta-2,4,6-triene**	$C_{10}H_{16}$	11.76	1120	1131	0.04±0.01	0.04±0.01	0.04±0.01	0.04±0.01
**2-Methyl-5-prop-1-en-2-ylcyclohexa-1,3-diene**	$C_{10}H_{12}$	11.96	1123	-	0.01±0.01	0.03±0.00	0.03±0.00	0.03±0.01
**Geranic acid**	$C_{10}H_{16}O_2$	15.64	1353	1355	0.09±0.04	0.10±0.03	0.11±0.05	0.12±0.04
**1-(2,5-dimethylphenyl) but-2-en-1-one**	$C_{12}H_{14}O$	16.46	1386	-	0.00±0.01	0.01±0.00	0.01±0.00	0.01±0.00
**2,6-Ditert-butyl-4-hydroxy-4-methylcyclohexa-2,5-dien-1-one**	$C_{15}H_{24}O_2$	17.26	1432	1478	0.01±0.01	0.02±0.01	0.02±0.00	0.01±0.00
**2,5,8-trimethyl-1,2-dihydronaphthalene**	$C_{13}H_{16}$	18.76	1544	-	0.00±0.00	0.01±0.00	0.01±0.00	0.01±0.00
**7,9-Di-tert-butyl-1-oxaspiro(4,5)deca-6,9-diene-2,8-dione**	$C_{17}H_{24}O_3$	22.86	1890	1916	0.01±0.01	0.03±0.01	0.02±0.00	0.02±0.00

[a] RT: Retention Time

[b] RI_{exp}: Experimental Retention Indices

[c] RI_{lit}: Retention Indices from literature (non-polar columns)

[d] References for RI_{lit}: [5,7,12,21,45–52]

[e] Concentration is in the order of magnitude of μg L^{-1}

Table S3. Identified metabolites using TMS derivatization before GC-MS analysis, their chemical formula, retention time, TMS derivative and their peak area ratios between 5th, 8th, 13th and the 1st day of fermentation.

A/A	Compound Name	Chemical Formula	RT	TMS derivative	General trend of metabolites during fermentation expressed as peak area (P.a.) ratios of each fermentation day with the 1st day			
	**ALCOHOLS-SUGAR ALCOHOLS**				**P. a. of 1st day**	**P.a. 5th/ P.a. 1st**	**P.a. 8th/ P.a. 1st**	**P.a. 13th/ P.a. 1st**
1	**1,3-Propanediol**	C3H8O2	8.46	2TMS	1DAPA$_{1}$[a]	1.3743	1.6433	1.6770
2	**2,3-Butanediol**	C4H10O2	9.07	2TMS	1DAPA$_{2}$	1.4779	2.0337	3.1670
3	**1,2,3-Butanetriol**	C4H10O3	14.78	3TMS	1DAPA$_{3}$	0.8656	1.4710	1.6818
4	**1,2-Butanediol**	C4H10O2	16.35	2TMS	1DAPA$_{4}$	1.1024	1.5093	1.9141
5	**Phenylethyl Alcohol**	C8H10O	13.15	TMS	1DAPA$_{5}$	2.2093	3.6277	3.3114
6	**Glycerol**	C3H8O3	14.42	3TMS	1DAPA$_{6}$	1.0920	1.4716	2.1705
7	**Diethylene glycol**	C4H10O3	14.73	2TMS	1DAPA$_{7}$	1.2457	1.3907	1.7031
8	**Threitol**	C4H10O4	17.92	4TMS	1DAPA$_{8}$	1.1650	1.2475	1.2858
9	***meso*-Erythritol**	C4H10O4	18.01	4TMS	1DAPA$_{9}$	1.1897	1.1952	1.2031
10	**1-Deoxypentitol**	C5H12O4	18.42	4TMS	1DAPA$_{10}$	1.1682	1.2164	1.5044
11	**Tyrosol**	C8H10O2	18.62	2TMS	1DAPA$_{11}$	1.0481	0.8904	1.2395
12	**Arabitol**	C5H12O5	20.59	5TMS	1DAPA$_{12}$	1.0997	1.1754	1.3484
13	**Mannitol**	C6H14O6	23.41	6TMS	1DAPA$_{13}$	1.1343	1.2132	1.4928
14	**Sorbitol**	C6H14O6	23.48	6TMS	1DAPA$_{14}$	1.1456	1.2228	1.2160
15	**Ribitol**	C5H12O5	23.49	5TMS	1DAPA$_{15}$	1.3182	1.6247	1.5761
16	***Scyllo*-Inositol**	C6H12O6	24.56	6TMS	1DAPA$_{16}$	0.9380	0.8307	0.8197
17	***Myo*-Inositol**	C6H12O6	25.18	6TMS	1DAPA$_{17}$	0.9130	0.9332	0.8310
18	**Dulcitol**	C6H14O6	27.46	6TMS	1DAPA$_{18}$	1.1785	1.2236	1.2099
	**ACIDS**							
19	**Lactic acid**	C3H6O3	8.714	TMS	1DAPA$_{19}$	1.2645	2.2790	3.5070
20	**Succinic acid**	C4H6O4	14.96	2TMS	1DAPA$_{20}$	1.0110	1.6692	2.0534
21	**Glyceric acid**	C3H6O4	15.37	3TMS	1DAPA$_{21}$	1.2361	1.0475	1.4904
22	**Citramalic acid**	C5H8O5	17.44	3TMS	1DAPA$_{22}$	1.2481	0.9697	1.5935
23	**Malic acid**	C4H6O5	17.68	3TMS	1DAPA$_{23}$	0.7515	0.6244	0.3269
24	**2-Methylglyceric acid**	C4H8O4	18.57	3TMS	1DAPA$_{24}$	1.2253	1.0847	1.3488
25	**Threonic acid**	C4H8O5	18.69	4TMS	1DAPA$_{25}$	1.0116	1.1767	1.1784
26	**Glutaric acid**	C5H8O4	18.75	2TMS	1DAPA$_{26}$	1.4295	0.7514	1.4938
27	**Xylonic acid**	C5H10O6	19.43	3TMS	1DAPA$_{27}$	1.1907	0.9799	1.2680
28	**Tartaric acid**	C4H6O6	19.63	4TMS	1DAPA$_{28}$	0.9280	0.7936	0.6208
29	**Gluconic acid**	C6H12O7	19.79	4TMS	1DAPA$_{29}$	0.9313	0.9352	1.1492
30	**Glucuronic acid**	C6H10O7	20.42	3TMS	1DAPA$_{30}$	0.8707	0.8310	0.8508
31	**Citric acid**	C6H8O7	21.64	4TMS	1DAPA$_{31}$	1.0294	1.2650	1.4802
32	**Galactaric acid**	C6H10O8	23.82	6TMS	1DAPA$_{32}$	0.8996	0.7864	0.5494
33	**Ribonic acid**	C5H10O6	24.30	4TMS	1DAPA$_{33}$	1.1731	1.2730	0.7503
34	**Palmitic Acid**	C16H32O2	24.36	TMS	1DAPA$_{34}$	1.1958	0.9954	0.7236
35	**Gluconic acid**	C6H12O7	24.46	6TMS	1DAPA$_{35}$	1.0305	0.7189	0.8407
36	**Caffeic acid**	C9H8O4	25.39	3TMS	1DAPA$_{36}$	1.0587	0.7740	0.6902
37	**Stearic acid**	C18H36O2	26.20	TMS	1DAPA$_{37}$	0.9490	0.5399	0.6501
	**AMINO ACIDS**							

**38**	***gamma*-Aminobutyric acid (GABA)**	C4H9NO2	13.41	3TMS	1DAPA$_{38}$	1.2467	0.9644	0.9892
**39**	**Proline**	C5H9NO2	14.65	2TMS	1DAPA$_{39}$	1.0757	1.2487	1.1863
**40**	**Glysine**	C2H5NO2	14.84	TMS	1DAPA$_{40}$	1.0289	0.8563	0.6295
**41**	**Serine**	C3H7NO3	23.41	TMS	1DAPA$_{41}$	0.83489	0.3288	0.2579
		**SUGARS**						
**42**	**Threose**	C4H8O4	17.82	3TMS	1DAPA$_{42}$	1.1247	1.5839	1.7325
**43**	**Arabinose**	C5H10O5	19.95	4TMS	1DAPA$_{43}$	1.0976	1.2513	1.8284
**44**	**Xylose**	C5H10O5	20.12	4TMS	1DAPA$_{44}$	1.0467	1.1906	1.8668
**45**	**Lyxose**	C5H10O5	20.33	4TMS	1DAPA$_{45}$	1.1070	1.2135	1.4435
**46**	**Methyl *alpha*-gluco-furanoside**	C7H14O6	21.41	4TMS	1DAPA$_{46}$	1.2870	1.1810	1.1451
**47**	**Allose**	C6H12O6	22.06	5TMS	1DAPA$_{47}$	1.1743	1.3678	1.5863
**48**	**Mannose**	C6H12O6	22.15	5TMS	1DAPA$_{48}$	1.2417	1.6948	2.1290
**49**	**Fructose**	C6H12O6	22.53	5TMS	1DAPA$_{49}$	0.7347	0.5248	0.1021
**50**	**Ribose**	C5H10O5	22.78	4TMS	1DAPA$_{50}$	0.9001	0.8498	0.7604
**51**	**Galactose**	C6H12O6	22.84	5TMS	1DAPA$_{51}$	1.1181	1.2835	1.4145
**52**	**Glucose**	C6H12O6	22.93	5TMS	1DAPA$_{52}$	0.3748	0.2133	0.0041
**53**	**Tallose**	C6H12O6	23.02	5TMS	1DAPA$_{53}$	1.0729	1.1529	1.4292
**54**	**Galactose oxime**	C6H13NO6	24.29	6TMS	1DAPA$_{54}$	1.2423	1.6336	1.7156
**55**	**Rhamnose**	C6H12O5	24.60	4TMS	1DAPA$_{55}$	1.1853	1.3276	1.9367
**56**	**2-O-Glycerol-*alpha*-ga-lactopyranoside**	C9H18O8	26.95	6TMS	1DAPA$_{56}$	1.0909	0.9800	0.9789
**57**	**Psicose**	C6H12O6	29.39	5TMS	1DAPA$_{57}$	1.0507	1.2073	1.2925
**58**	**Sucrose**	C12H22O11	29.47	8TMS	1DAPA$_{58}$	1.0120	0.8745	0.7386
**59**	**Lactose**	C12H22O11	29.66	8TMS	1DAPA$_{59}$	0.8947	0.8505	0.9103
**60**	**Maltose**	C12H22O11	29.78	8TMS	1DAPA$_{60}$	0.9438	0.7933	0.5600
**61**	**2-*alpha*-Mannobiose**	C12H22O11	29.84	8TMS	1DAPA$_{61}$	0.9098	0.9200	1.0463
**62**	**Cellobiose**	C12H22O11	29.87	8TMS	1DAPA$_{62}$	1.0307	0.9705	1.0930
**63**	***beta*-Gentiobiose**	C12H22O11	30.05	8TMS	1DAPA$_{63}$	0.9352	0.9004	1.0806
		**OTHERS**						
**64**	**1-(3-Methylbutyl)-2,3,4,6-tetramethylben-zene**	C15H24	9.96	2TMS	1DAPA$_{64}$	0.8901	0.8352	0.8378
**65**	**Hydroxylamine**	H3NO	10.22	3TMS	1DAPA$_{65}$	1.1578	0.8487	1.1572
**66**	**Cadaverine**	C5H14N2	14.10	4TMS	1DAPA$_{66}$	1.0134	0.8568	0.7591
**67**	**Glucuronic acid *gamma*-lactone**	C6H8O6	21.84	3TMS	1DAPA$_{67}$	1.0790	1.4463	0.8637
**68**	**Allonic acid *gamma*-lac-tone**	C6H10O6	23.99	4TMS	1DAPA$_{68}$	1.4251	1.8538	0.6959
**69**	**1-Monopalmitin**	C19H38O4	28.54	2TMS	1DAPA$_{69}$	0.8835	0.5103	0.6841

[a]1DAPA: 1st day Average (11 tanks) Peak Area

Table S4. *P*-values, p(corr) and VIP values of the statistically significant volatile metabolites of the discrimination between 1st and 13th day of fermentation

Compound	*p*-value	p(corr)	VIP
**Ethyl octanoate**	5.34E-09	0.9379	4.0341
**2-Phenylethanol**	8.49E-06	0.7767	3.5865
**Isoamyl alcohol**	9.01E-08	0.8622	3.4356
**Octanoic acid**	0.0003	0.6889	3.3633
**Ethyl Decanoate**	1.92E-06	0.8858	3.0607
**Isoamyl acetate**	7.42E-05	0.7490	3.0337
**Phenethyl acetate**	0.0001	0.7262	2.5737
**Ethyl Acetate**	1.21E-11	0.9628	2.3877
**Ethyl hexanoate**	3.02E-07	0.8442	1.9489
**2-Methylbutan-1-ol**	7.39E-08	0.8600	1.9266
**Ethyl Dodecanoate**	0.000148	0.8064	1.5869
**Isobutyl lactate**	1.05E-13	0.9868	1.5405
**Linalool**	0.0043	-0.5795	1.3124
**2,3-Butanediol**	9.1E-15	0.9898	1.2243
**2-Methylpropan-1-ol**	1.09E-10	0.9461	1.1444
**Butanoic acid**	0.0002	0.6851	1.0305
**Hexanoic acid**	0.0002	0.6772	1.0159
**Ho-trienol**	1.09E-05	-0.8126	0.9883
***trans*-2-Pinanol**	1.96E-09	0.9074	0.9831
**Nerol**	1.28E-08	0.8820	0.9668
***alpha*-Terpineol**	0.0130	-0.5238	0.8206
**2-Methylbutyl acetate**	0.0001	0.7147	0.8196

Table S5. *P*-values, p(corr) and VIP values of the statistically significant volatile metabolites of the discrimination between 1st and 5th day of fermentation

Compound name	*p*-value	p(corr)	VIP
**Octanoic acid**	1.83E-05	0.7813	3.9640
**2-Phenylethanol**	1.44E-05	0.8033	3.8710
**Ethyl octanoate**	1.56E-06	0.8288	3.4968
**Isoamyl alcohol**	1.00E-05	0.7903	3.2578
**Phenethyl acetate**	1.46E-05	0.8031	3.1062
**Isoamyl acetate**	2.93E-05	0.7514	2.9650
***n*-Decanoic acid**	0.0013	0.5885	2.6667
**Ethyl Decanoate**	0.0003	0.7033	2.4058
**Ethyl hexanoate**	1.04E-06	0.8172	2.0694
**2-Methylbutan-1-ol**	1.67E-05	0.7837	1.8900
**Ethyl Acetate**	2.92E-07	0.8688	1.8827
**Butanoic acid**	7.3E-07	0.8772	1.4219
**Hexanoic acid**	7.28E-07	0.8769	1.4133
**Linalool**	0.0077	0.5626	1.4079
**Hexyl acetate**	0.0074	0.5650	1.0924
**Isobutyl lactate**	3.25E-07	0.8709	1.0038
**Ethyl Dodecanoate**	3.3E-05	0.7507	0.9806
***alpha*-Terpineol**	0.0044	0.5967	0.9519
**2,4-Ditert-butylphenol**	0.0023	0.6128	0.9180
**Nerol**	3.02E-07	0.8698	0.9129
**2-Methylbutyl acetate**	3.51E-05	0.7560	0.9014
**2-Methylpropan-1-ol**	1.82E-06	0.8452	0.8909
***trans*-2-Pinanol**	6.96E-08	0.8904	0.8718

Table S6. *P*-values, p(corr) and VIP values of the statistically significant volatile metabolites of the discrimination between 5th and 8th day of fermentation

Compound name	*p*-value	p(corr)	VIP
**Ethyl octanoate**	0.0021	0.6153	3.7162
***n*-Decanoic acid**	0.0064	-0.5516	3.1940
**Isoamyl acetate**	0.0395	0.4162	3.1573
**Linalool**	9.38E-05	-0.8370	2.9754
**Isoamyl alcohol**	0.0482	0.5385	2.8287
**Ethyl Acetate**	0.0009	0.7032	2.6141
***alpha*-Terpineol**	1.62E-05	-0.8475	2.1641
**Isobutyl lactate**	3.34E-07	0.9239	2.0342
**2,3-Butanediol**	9.68E-07	0.9204	1.6907
**Ethyl Dodecanoate**	0.0149	0.5770	1.6698
**Geraniol**	3.92E-05	-0.8394	1.3836
**Ho-trienol**	0.0001	-0.8139	1.3656
**2-Methylpropan-1-ol**	0.0015	0.8164	1.2200
***beta*-Damascenone**	0.0002	-0.7539	1.1977
***trans*-2-Pinanol**	0.0003	0.7367	1.0594
**Hexanoic acid**	0.0308	-0.5336	0.9961
**Butanoic acid**	0.0327	-0.5295	0.9883
**Nerol**	0.0090	0.5796	0.8825

Table S7. *P*-values, p(corr) and VIP values of the statistically significant volatile metabolites of the discrimination between 8th and 13th day of fermentation

Compound name	*p*-value	p(corr)	VIP
**Octanoic acid**	0.0199	-0.8677	5.0460
***n*-Decanoic acid**	0.0191	-0.8796	3.5688
**Linalool**	0.0065	-0.8669	2.5720
**Hexanoic acid**	0.0282	-0.6259	1.4608
**Butanoic acid**	0.0272	-0.6308	1.4587
**Hexyl acetate**	0.0473	-0.6702	1.4080
**Ho-trienol**	0.0069	-0.7417	1.0507
**Geraniol**	0.0403	-0.8041	1.0458

Table S8. *P*-values, p(corr) and VIP values of the statistically significant non-volatile polar metabolites of the discrimination between 1st and 13th day of fermentation

Compound name	*p*-value	p(corr)	VIP
**Fructose**	0.0010	-0.6433	2.5134
**Glucose**	0.0009	-0.6433	2.5113
**Mannose**	0.0009	-0.6548	2.3871
**Tallose**	2.68E-05	-0.9903	2.3420
**Galactose**	0.0021	-0.6282	1.9671
**Ribose**	0.0317	-0.7950	1.7780
**Glucose**	2.65E-05	-0.9786	1.3907
**Threose**	2.49E-05	-0.9778	1.3875
**Allose**	4.20E-05	-0.9825	1.2409

Table S9. *P*-values, p(corr) and VIP values of the statistically significant non-volatile polar metabolites of the discrimination between 1st and 5th day of fermentation

Compound name	*p*-value	p(corr)	VIP
**Glucose**	0.0014	-0.6800	2.9002
**Mannose**	0.0014	-0.6916	2.7727
**Galactose**	0.0026	-0.6656	2.3586
**Fructose**	0.0001	-0.6142	2.3351
**Ribose**	0.0229	-0.6455	1.7883
**Xylose**	0.0112	-0.5635	1.0894
**Allose**	0.0127	-0.5692	1.0331

Table S10. *P*-values, p(corr) and VIP values of the statistically significant non-volatile polar metabolites of the discrimination between 5th and 8th day of fermentation

Compound name	*p*-value	p(corr)	VIP
**Glucose**	1.06E-05	-0.6673	3.4498
**Galactose**	2.53E-05	-0.6976	3.3014
**Talose**	2.77E-05	-0.6231	3.3005
**Threose**	1.71E-05	-0.6438	1.8916
**Mannose**	1.83E-05	-0.6352	1.8654
**Allose**	2.14E-05	-0.6193	1.7100

Table S11. *P*-values, p(corr) and VIP values of the statistically significant non-volatile polar metabolites of the discrimination between 8th and 13th day of fermentation

Compound name	*p*-value	p(corr)	VIP
**Fructose**	0.0008	-0.6974	6.5981
**Glycerol**	0.0100	-0.5834	2.8347
**Galactose**	0.0549	-0.5298	2.6206
**Glucose**	0.0582	-0.5065	2.4166
**Tartaric acid**	0.0027	0.5084	1.0978
**Allose**	0.0538	-0.5012	1.0630
**Malic acid**	0.0056	0.5874	1.0162
**Succinic acid**	0.0022	0.6149	0.9618

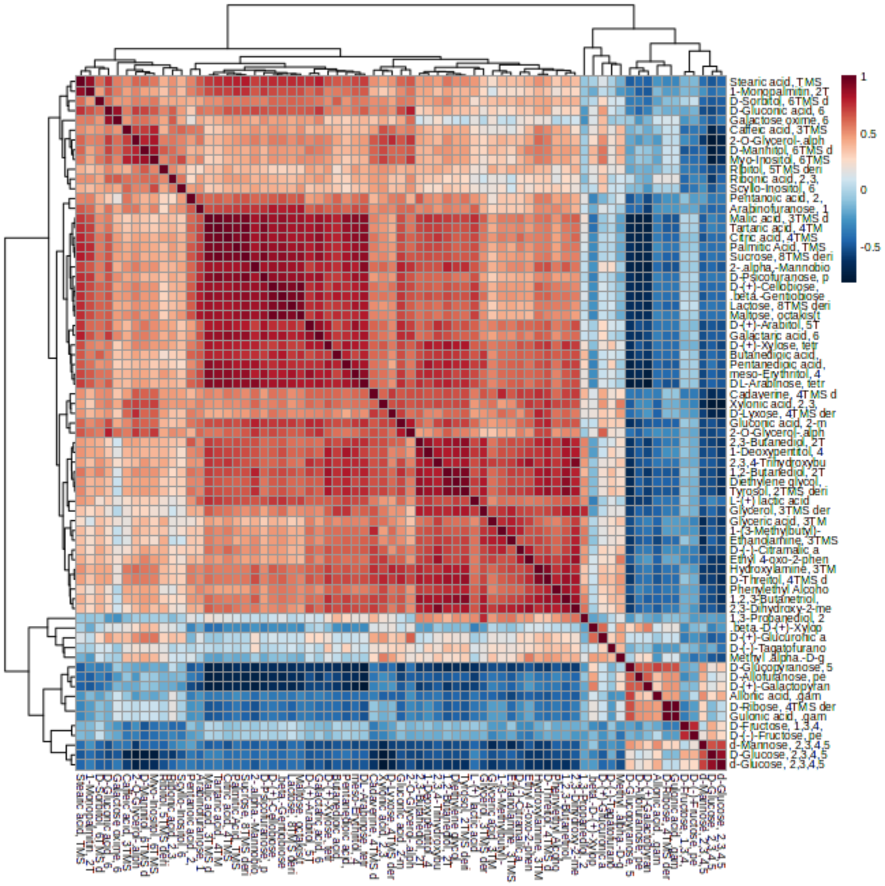

Figure S1. Correlation heatmap of the identified compounds using the derivatization method

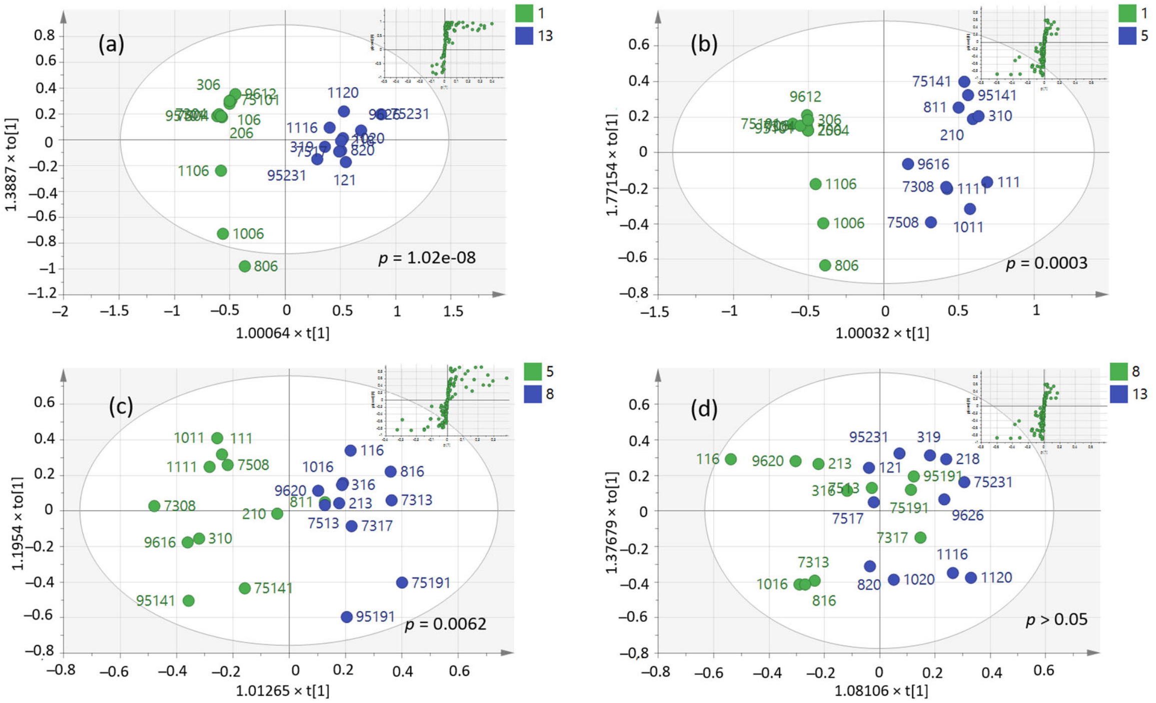

Figure S2. OPLS-DA models and their corresponding S-plots between (a) 1-13, (b) 1-5, (c) 5-8 and (d) 8-13 days of fermentation, analyzing with the SPME method

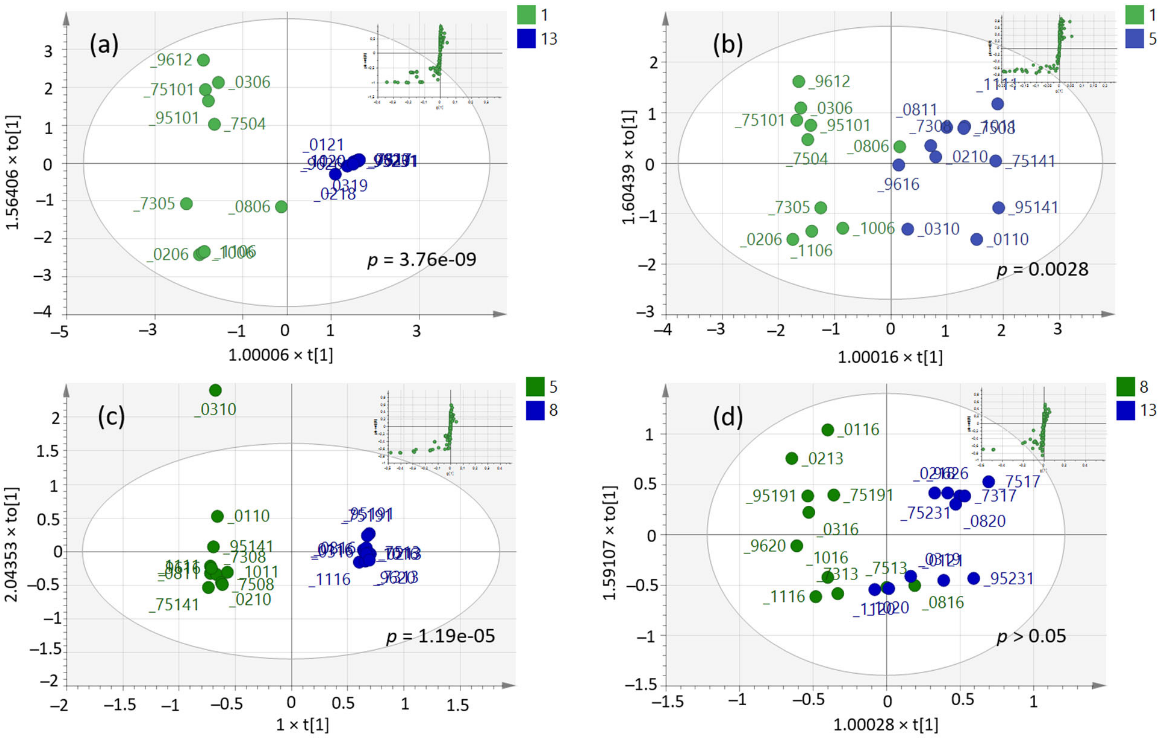

Figure S3. OPLS-DA models and their corresponding S-plots between a) 1-13, b) 1-5, c) 5-8 and d) 8-13 days of fermentation, analyzing with the derivatization method
